# The LAMP-CRISPR-Cas13a technique for detecting the CBASS mechanism of phage resistance in bacteria

**DOI:** 10.3389/fmicb.2025.1550534

**Published:** 2025-03-24

**Authors:** Concha Ortiz-Cartagena, Patricia Fernández-Grela, Lucia Armán, Lucía Blasco, Daniel Pablo-Marcos, Inés Bleriot, Laura Fernández-García, Clara Ibarguren-Quiles, Felipe Fernández-Cuenca, Antonio Barrio-Pujante, Belén Aracil, Jorge Calvo-Montes, María Tomás

**Affiliations:** ^1^Departamento de Microbiología-Hospital A Coruña (HUAC), Grupo de Microbiología Traslacional y Multidisciplinar (Micro-TM), A Coruña, Spain; ^2^Grupo de Estudio de Mecanismos de Acción y Resistencia a los Antimicrobianos (GEMARA) en nombre de la Sociedad Española de Enfermedades Infecciosas y Microbiología Clínica (SEIMC), Madrid, Spain; ^3^Microbiology Service, University Hospital Marqués de Valdecilla—IDIVAL, Santander, Spain; ^4^Microbiology Service, University Hospital Virgen Macarena-IBIS, Seville, Spain; ^5^CIBER de Enfermedades Infecciosas (CIBERINFEC), Instituto de Salud Carlos III (ISCIII), Madrid, Spain; ^6^Laboratorio de Referencia e Investigación de Resistencias Antibióticas e Infecciones Sanitarias, Centro Nacional de Microbiología, ISCIII, Majadahonda, Spain; ^7^MEPRAM, Proyecto de Medicina de Precisión contra las resistencias Antimicrobianas, ISCIII, Majadahonda, Spain

**Keywords:** phage resistance, CBASS systems, CRISPR, Cas13a, Purification-free

## Abstract

**Introduction:**

Antimicrobial resistance (AMR) is a major public health threat, driving the need for alternative treatments such as phage therapy. However, bacterial defense mechanisms, often regulated by the quorum sensing (QS) network and encoded in genomic islands (GIs), can generate phage-resistant mutants. Understanding these resistance mechanisms is essential for optimizing phage therapy.

**Methods:**

This study analyzed 48 *Klebsiella pneumoniae* strains to identify pathogenicity islands (PAIs) containing anti-phage defense (APD) proteins. We constructed a knockout strain lacking the cyclase gene from the type II CBASS defense systems present in PAIs to investigate QS regulation and its role in cell viability. The LAMP-CRISPR-Cas13a technique was used to confirm gene knockout and to detect the main cyclase in type I CBASS systems, i.e., APECO1.

**Results:**

A total of 309 pathogenicity islands (PAIs), containing 22.1% of anti-phage defense (APD) proteins, were identified. Type I and II CBASS APD systems were also detected in the genome of the 48, *K. pneumoniae* strains, and only two type II CBASS systems were located in PAIs. Alluding to these defense mechanisms, the QS revealed to be involved in the regulation of the type II CBASS systems contained in PAIs. Finally, the LAMP-CRISPR-Cas13a technology successfully detected the main cyclases habored in type I and II CBASS systems, respectively.

**Discussion:**

The study findings highlight the regulatory role of the QS network in APD systems. Notably, this is the first study to develop an innovative biotechnological application for the LAMP-CRISPR-Cas13a rapid-technique (<2 h), thereby helping to optimize phage therapy by detecting bacterial resistance mechanisms and predicting the potential inefficacy of therapeutic phages and thus improving patient prognosis.

## Introduction

Antimicrobial resistance (AMR) is one of the major current global threats to public health, and it has been predicted that as many as 10 million people could die annually as a result of AMR by 2050 (Murray et al., 2022; Jonas et al., 2017; Tang et al., 2023). Worryingly, the 2022 Global Antimicrobial Resistance and Use Surveillance System (GLASS) report raised concerns about extremely high resistance rates among prevalent bacterial pathogens (Ajulo and Awosile, 2024). In this context, carbapenem-resistant *Klebsiella pneumoniae* is highlighted as a key pathogen against which common antibiotics are no longer effective. This critical situation has led to the increased administration of last-resort drugs, with resistance to these drugs increasing rapidly (Reyes et al., 2019). *K. pneumoniae* is included in the Bacterial Priority Pathogens List (BPPL) compiled by the World Health Organization (WHO), implying that measures for prevention and appropriate treatment of infections are crucial (WHO, 2024).

Regarding the scarcity of effective treatments for AMR infections, due to the emergence of resistance to all antibiotics used in clinical settings, therapeutic innovations such as phage therapy are becoming more important (Bleriot et al., 2023a). Phage therapy, the use of bacteriophages (phages, i.e., viruses that infect bacteria) to treat bacterial infections, has several advantages over other alternative treatments (Lin et al., 2017; Pacios et al., 2020; Singh et al., 2023). However, contact between bacteria and phage leads to the so-called phage-host arms race, and one of the greatest disadvantages of phage therapy is the frequent and rapid generation of phage-resistant bacterial mutants during treatment (Georjon and Bernheim, 2023; Wang and Zhang, 2023). Anti-phage defense (APD) mechanisms in bacteria are mainly based on (i) adsorption resistance, (ii) blockage of the phage infection once adsorbed, (iii) elimination of the viral genetic material, i.e., restriction-modification (RM) and clustered regularly interspaced short palindromic repeat (CRISPR)-associated protein (CRISPR-Cas) systems, and (iv) other mechanisms, including toxin–antitoxin (TA) systems and abortive infection (Abi) systems such as cyclic oligonucleotide-based anti-phage signaling systems (CBASS) (Wang and Zhang, 2023; Fillol-Salom et al., 2022).

APD systems are commonly contained in clusters of genes within a bacterial genome, known as genomic islands (GIs). These genes appear to have been acquired through horizontal gene transfer (HGT), which rapidly spreads genes that improve bacterial fitness and adaptation (Audrey et al., 2023; Dobrindt et al., 2004; Ramsay et al., 2023). The genes harbored in GIs are classified as pathogenicity islands (PAIs), metabolic islands (MIs), symbiotic islands (SIs), and resistance islands (RIs) (Ramsay et al., 2023; Leamon and Rothberg, 2009). Although APD systems are frequently clustered together in bacterial genomes in defense islands (DIs), recent findings have revealed the essential role of PAIs in holding and spreading bacterial defense systems. This highlights the importance of studying APD proteins to improve our understanding of bacterial immunity and to develop therapeutic strategies (Fillol-Salom et al., 2022; Doron et al., 2018; Oliveira et al., 2016). Some APD systems harbored by PAIs, such as CBASS and RM systems, protect bacteria from phage infections and also seem to influence bacterial competition and HGT, ultimately shaping microbial communities (Doron et al., 2018; Oliveira et al., 2016).

Furthermore, phages themselves can mobilize PAIs that contain immune systems, as shown in *Staphylococcus aureus*, in which phage-inducible chromosomal islands (PICIs) have been shown to facilitate the spread of APD genes (Humphrey et al., 2021). This suggests that PAIs act as key genetic reservoirs for bacterial defense, reinforcing the importance of studying their role in the evolution and adaptation of pathogens, as well as their impact on the efficacy of phage therapy (Fillol-Salom et al., 2022; Humphrey et al., 2021).

Among the APD systems, the CBASS systems are a family of defense systems composed of an oligonucleotide cyclase, which generates signaling cyclic oligonucleotides in response to phage infection. These cyclic oligonucleotides activate an effector that promotes cell death before phage replication is completed, therefore preventing the spread of phages to nearby cells (Millman et al., 2020). Diverse CBASS defense systems have been identified and are classified according to the composition of the CBASS operon, the activity of the effector protein, and the signaling molecule produced by the oligonucleotide cyclase. Type I and II systems are the most common. The type I CBASS system consists of an oligonucleotide cyclase and an effector protein, the so-called minimal configuration, and the effector activity is mainly based on the formation of pores in the membrane. In addition to the cyclase–effector framework, the type II CBASS system has two ancillary genes (*cap2* and *cap3*) that are involved in expanding the range of phages against which it acts, and the effector protein is usually a phospholipase (Millman et al., 2020).

Importantly, many bacteria control the expression of several of these defense mechanisms, such as CBASS systems, through the quorum sensing (QS) network, which is defined as a cellular communication process mediated by signaling molecules called autoinducers (AIs) (Barrio-Pujante et al., 2024; Díaz, 2017). The QS of *K. pneumoniae* mainly relies on the use of autoinducer-2 (AI-2). AI-2 consists of a group of 4,5-dihydroxy-2,3-pentanedione (DPD) derivatives that can rapidly convert to one another, are involved in interspecies communication, and are capable of detecting other AIs in the medium such as AI-1 (exogenous N-Acyl homoserine lactones or AHLs) (Díaz, 2017; Pacheco et al., 2021). In a previous study, the inhibition of QS led to a reduction in the expression of CBASS proteins, which meant an improvement in phage infection, bringing to light the role that QS plays in controlling bacterial APD (Barrio-Pujante et al., 2024). Under this premise, the strategy of counteracting CBASS systems may be a useful way of enhancing the therapeutic outcomes of phages in clinical settings.

Detection of the anti-phage systems harbored in a targeted clinical strain can be used to overcome bacterial resistance to phage, hence enabling the application of personalized phage therapy treatments that improve patient prognosis. In this context, CRISPR-Cas systems are potentially promising tools, as they have yielded successful results regarding the development of detection protocols with high levels of sensitivity and specificity (Kellner et al., 2019; Chen et al., 2018; Zahra et al., 2023). These diagnostic techniques have previously been used to detect several microorganisms and also resistance genes (Ortiz-Cartagena et al., 2022, 2023; Curti et al., 2020; Wu et al., 2021).

In this study, we analyzed the APD genes contained in the PAIs detected in the genome of 48 *K. pneumoniae* isolates. We also conducted an epidemiological study of the CBASS systems located in these strains, focusing on the QS-based regulation of type II CBASS APD system in a carbapenemase-producing *K. pneumoniae* clinical strain. Finally, we broadened the range of biotechnological applications of CRISPR-Cas technology, by applying our previously developed diagnostic protocol based on the CRISPR-Cas13a system to detect anti-phage resistance genes in bacteria (Ortiz-Cartagena et al., 2022, 2023).

## Materials and methods

### *In silico* analysis

Genomes in contigs of 48 *K. pneumoniae* clinical isolates (Table 1), previously sequenced by Illumina MiSeq (BioProject codes listed in Table 1; Bleriot et al., 2023b; Grundmann et al., 2017), were studied. Contigs were assembled through an aleatory number of “Ns” to compress the genomes into single sequences with the Linux OS. In order to obtain files in GenBank format, the genomes were then reannotated using the PROKKA software tool (Seemann, 2014), also with the Linux OS.

**Table 1:** Hospital of origin of the 48 *K. pneumoniae* clinical strains.

**Hospitals**	**National Center of Microbiology (Madrid, Spain)^a^**	**Virgen Macarena University Hospital (Seville, Spain)^b^**
Strain	1ST405OXA48	K2535S
	2ST15VIM1	K2551S
	3ST11OXA245	K2597S
	4ST437OXA245	K2691S
	5ST16OXA48	K2707S
	6ST101KPC2	K2715S
	7ST147VIM1	K2783S
	8ST11VIM1	K2791S
	9ST846OXA48	K2982S
	10ST340-VIM1	K2983S
	12ST13OXA48	K2984S
	13ST512KPC3	K2986S
	14ST15OXA48	K2989S
	15ST11OXA48	K2990S
	16ST258KPC3	K3318S
	17ST899OXA48	K3320S
	18ST974OXA48	K3321S
	SCISP2CS	K3322S
	SCISP4CS	K3323S
		K3324S
		K3325S
		K3416S
		K3509S
		K3571S
		K3572S
		K3573S
		K3574S
		K3575S
		K3579S
		K3667S

^a^BioSample code of complete genomes included in the European BioProject PRJNA565865.

^b^BioSample code of complete genomes included in the European BioProject PRJEB10018.

### Identification and analysis of genomic islands

In order to predict the GIs harbored in the genomes from 48 *K. pneumoniae* clinical isolates, the genomes were analyzed using Genomic Island Prediction (GIPSy) software (Soares et al., 2016) in Windows and applying standard values in steps 2–7, following the GIPSy manual (Version 1.1.2). The GIs were classified into PAIs, MIs, SIs, and RIs by using GIPSY software, with the genomes in GenBank format files as input and the genome of *K. pneumoniae* strain MGH78578 (GenBank: NC_009648) as the reference genome. The sequences of the predicted PAIs were extracted from their respective genomes; the “Ns” were removed, and the sequences were annotated using the PROKKA software tool, in all cases with the Linux OS. The fasta files of these protein sequences were analyzed, using the HHMER (Finn et al., 2011) and HHPred (Söding et al., 2005) servers, along with the NCBI conserved domains database (Wang et al., 2023), to identify and thus manually classify the proteins contained in them, as follows: (i) metabolic proteins; (ii) defense, resistance, and virulence proteins (DRV); (iii) DNA metabolism proteins; (iv) MGE; and (v) unknown proteins. APD genes were also detected by PADLOC, a web server for the identification of antiviral defense systems in microbial genomes, using GenBank format files as input; the defense sequences were then analyzed using HHMER and HHPred servers, as previously described (Payne et al., 2022). For the servers and database used in this bioinformatical analysis, standard values and default parameters were applied.

### Cyclase gene knockout strain

#### Strains, plasmids, and reagents

The *K. pneumoniae* strain 10ST340-VIM1 was selected from the bacterial strain collection (Table 1), to construct a knockout of the cyclase gene (10ST340-VIM1Δcyclase) present in a type II CBASS system, by a protocol using the *Escherichia coli* λ-Red system (Huang et al., 2014). This protocol is based on homologous recombination mediated by the λ-Red system of the λ phage that is used to eliminate specific genes in *E. coli* strains. In order to adapt it to *K. pneumoniae*, it was applied with some modifications. The homologous regions, required for the recombination step in the target genome, were of length 300 bp, and therefore, the length of the knockout cassette ends was increased to 300 bp. Plasmids and primers used for this goal are listed in Table 2.

**Table 2:** Primers and plasmids used to construct the 10ST340-VIM1Δcyclase *K. pneumoniae* strain.

**Name**	**Sequence 5^′^-3^′^**
**Primer**	
F_300 up	GGCACTGTTTTCAGGGAGGAG
B_300 up	GAGGCTGGCGGGAACTTCGAAGTTCCTATACTTTCTAGAGAATAGGAACTTCGAACTGCAGGTCGACGGATCCCCGGAATCATGAATTTGCCC TCACATCAGAATC
F_middle	GTAGGTGTTTTTAGGCTTTCATGTACATGATATTGATTCTGATGTGAGGGCAAATTCATGATTCCGGGGATCCGTCGACC
B_middle	GTGATACTGTTCAGCAAGGGTAAGGCGCCGATTAGGCAAAAAGACATTCATGGTCAGTTATGTAGGCTGGAGCTGCTTC
F_300 down	TGGCTGAGCTCATAAGTTCCTATTCCGAAGTTCCTATTCTCTAGAAAGTATAGGAACTTCGAAGCAGCTCCAGCCTACATAACTGACCATGAAT GTCTTTTTG
B_300 down	TTCCGAGTCAGTCGCGTATGT
F_Conf_Int	AGGTGGGAAAGAGCGGATTG
B_Conf_Int	AGCTCAAGGACATCCAGCAGT
F_Conf_Ext	CGCAGCCAAACACAGATTCC
B_Conf_Ext	GGAGGAGCGTATTCAATCCG
**Name**	**Application**	**Resistance**
**Plasmid**		
pMDIAI	Knockout cassette construction	Apramycin
pACBRS-Hyg	Harbors λ red system	Hygromycin
pFLP-Hyg	Harbors FLP recombinase	Hygromycin

Bacterial cultures were grown in Luria-Bertani (LB) broth (10 g/L tryptone, 5 g/L yeast extract, and 10 g/L NaCl) or on LB agar (LB broth supplemented with 20 g/L of agar). Hygromycin and apramycin were used to select transformant strains at concentrations of 100 and 50 μg/mL, respectively.

The cyclase gene was deleted from the *K. pneumoniae* 10ST340-VIM1 strain by the above-mentioned method (Huang et al., 2014). Briefly, the knockout cassette containing the apramycin resistance gene was electroporated into the *K. pneumoniae* 10ST340-VIM1 strain already transformed with plasmid pACBSR-Hyg, which expresses the λ-Red system and a hygromycin selection marker. Bacteria were incubated overnight on LB agar supplemented with apramycin, and correct insertion of the knockout cassette was confirmed by PCR (F_300up and B_Conf_Int). The *K. pneumoniae* 10ST340-VIM1 derivative colonies were grown on LB agar with apramycin (50 μg/mL), at 37°C for 3 days, before being incubated on LB agar with apramycin (150 μg/mL) and LB agar with hygromycin (100 μg/mL), overnight at 37°C, to test colonies for loss of the helper plasmid pACBSR-Hyg. For removal of resistance markers, positive colonies were electroporated with pFLP-Hyg and grown overnight at 30°C. The mutant colonies were then incubated at 43°C to remove the apramycin resistance from the inserted knockout cassette. Apramycin-susceptible mutant colonies were selected to confirm deletion of the cyclase gene by PCRs (F_300up/B_300down and F_Conf_Int/B_Conf_Int) and Sanger sequencing (Macrogen^®^).

### Induction of expression of the CBASS by QS

Expression of effector I was quantified in the presence of 20 μM of AHL C6-HSL in both the 10ST340-VIM1 and 10ST340-VIM1Δcyclase *K. pneumoniae* strains. For this purpose, 40 μL of an overnight culture of the corresponding *K. pneumoniae* strain was inoculated in 4 mL flasks of LB-broth containing 20 μM C6-HSL (two biological samples of each strain). The flasks were then incubated at 37°C, with shaking at 180 rpm, until the optical density measured at a wavelength of 600 nm (OD_600_) reached 0.3.

Duplicate aliquots of 1 mL were removed from each flask for subsequent RNA extraction with the High Pure RNA Isolation Kit (Roche). Extraction was conducted following the manufacturer's instructions. The RNA extracted from each sample was measured with a Nanodrop spectrophotometer (NanoDrop Technologies) and adjusted to 50 ng/μL with nuclease-free water for use in qRT-PCR (LightCycler^®^ 480). Specific primers and the corresponding Universal ProbeLibrary (UPL) probe for both the *effector I* and *recA* (Gomes et al., 2018) genes were designed using ProbeFinder software (Roche) and are listed in Table 3. This assay was conducted with the LightCycler^®^ 480 Control Kit (Roche). Student's *t*-test (GraphPad Prism 9.0.0) was used to determine any statistically significant differences (*p*-value < 0.05) in gene expression.

**Table 3:** Primers and probes used for *effector I* and *recA* genes in the qRT-PCRs.

**Primer name**	**Sequence 5^′^-3^′^**	**Probe**	**References**
***Effector I*** **gene**			
Fwd_CBASS _Effector1	CGAACGCTGGGAGCATATCT	152	This study
Rv_CBASS _Effector1	CGATTTCGTCCAATTCTCCGG	152	This study
***RecA*** **gene**			
Fwd_RecA	TCGGTCAGGGTAAAGCGAAC	149	This study
Rv_RecA	TGGCTTCGCATCCTGATTGT	149	This study

### Cell viability in the presence of overexpression of CBASS

Cell proliferation and cell viability levels in both 10ST340-VIM1 and 10ST340-VIM1Δcyclase *K. pneumoniae* strains cultured in the presence of 20 μM of C6-HSL (to overexpress the CBASS) were determined by an assay based on the WST-1 reagent, a red tetrazolium salt that turns yellow when cleaved to formazan by mitochondrial dehydrogenases (WST-1 Assay Protocol for Cell Viability, MERCK^®^). Briefly, overnight cultures of the corresponding *K. pneumoniae* strains were diluted 1:100 in LB broth (two biological samples of each strain) and the flasks were incubated at 37°C, with shaking at 180 rpm, until the OD_600_ reached 0.2. The cultures were then diluted to OD_600_ 0.02 and added to the wells of 96-well plates and made up to a final volume of 100 μL supplemented with 20 μM of C6-HSL. The plates were incubated at 37°C, with shaking at 180 rpm for 24 h, and the OD_600_ was again read in a microplate reader (NanoQuant). Dilutions were made in new 96-well plates to adjust the OD_600_ to 0.02, and 10 μL of WST-1 reagent was then added to the plates, which were incubated at 37°C in the absence of light for 1.5 h. Cell proliferation and viability were quantified at OD_480_. Student's *t*-test (GraphPad Prism 9.0.0) was used to determine any statistically significant differences (*p*-value < 0.05) in proliferation levels. In this assay, negative controls consisted of LB without strains nor C6-HSL supplementation.

### Detection of CBASS by CRISPR-CAS13

#### Nucleic acid preparations

The gene sequences for cyclase APECO1 and type II CBASS system cyclase of each *K. pneumoniae* strain were assembled using Prokka software (Seemann, 2014). The target sequences were analyzed *in silico* by aligning each of the sequences of both kinds of cyclases using MEGA11 software (Tamura et al., 2021). For both types of detection, crRNA molecules were designed to target conserved regions, preferably targeting a C/G nucleotide for crRNA-Cas13a complex stability (Table 4). Thus, three pairs of LAMP primers were designed using PrimerExplorer V5 software (F3/B3, FIP/BIP, Floop/Bloop) in order to amplify the regions containing the targeted sequences (Table 4). The FIP LAMP primers included the T7 polymerase promoter in their sequences for the subsequent transcription step. The RNA reporter molecules, designed for signal amplification, harbor a single isomer derivative of fluorescein modification (FAM) at the 5′ extreme and a biotin molecule at the 3′ extreme.

**Table 4:** Primers, crRNA, and reporter molecules used to detect LAMP-CRISPR-Cas13a.

**Name**	**Sequence 5^′^-3^′^**	**Gene position**
**LAMP primer**		
F3_cyclase_I	CCTGTTTTTGAACAAGAGGA	370–389
B3_cyclase_I	TGTATACCAGAGTATCAATCAGA	564–586
FIP^a^_cyclase_I	GTCATTTCAGCTCTGGGCTTCGAAATTAATACGACTCACTATAGGGTGTAGTTATAAATATCCTGACACG	-
BIP_cyclase_I	TAGCAACCTCCGTCGGCTATAGCCCTCCCATAGCTAAG	-
LOOPF_cyclase_I	TTCCAACTGCCACCGTTTTT	415–434
LOOPB_cyclase_I	GTAAAATGATAAGGGCATGGCGTA	512–535
F3_cyclase_II	GACTTGGGTTATAACTCTTCAG	433–454
B3_cyclase_II	GGTTCAGAAGTTTCTTACGG	636–655
FIP^a^_cyclase_II	CAGGGTTTGATTCCTTCCAATCAGAAATTAATACGACTCACTATAGGGAGCACAACCATCCTCTCT	-
BIP_cyclase_II	TTACCGATATTTGTAAGGCTTGAGGTATGATCGGGTAATGGCC	-
LOOPF_cyclase_II	AGACGCGAATCAGGAACCA	473–491
LOOPB_cyclase_II	GAAAGCTTCATCAAATCAGCGGCAG	589–613
**crRNA**		
crRNA^b^_cyclase_I	gauuuagacuaccccaaaaacgaaggggacuaaaacUUUGUUUGCGUCAAGGUUAUUUAUAGCU	462–489
crRNA^b^_cyclase_II	gauuuagacuaccccaaaaacgaaggggacuaaaacAUGUUAUCAAACCAUGUCGCAUAACCUG	518–737
**Reporter**		
Reporter	FAM-UUUUUU-Biotin	-

All primers were supplied by IDT, and the reporter was supplied by GenScript.

^a^Underlined letters indicate the T7 promoter sequence.

^b^Lower-case letters indicate the scaffold sequence.

#### LAMP-CRISPR-Cas13a protocol

The LAMP-CRISPR-Cas13a detection protocol, previously described by our research group (Ortiz-Cartagena et al., 2022, 2023), was used to detect the strains harboring type I and type II CBASS systems from among the 48 *K. pneumoniae* isolates. In order to identify type I CBASS systems, the LAMP-CRISPR-Cas13a technique was targeted to the cyclase APECO1 gene, located in several type I CBASS systems; the presence of type II CBASS systems was determined by identifying the cyclase gene contained in both type II CBASS systems detected in the PAIs. For this purpose, genomic DNA was extracted from the respective strains by the proteinase K-heat inactivation protocol (PK-HID) (Genoud et al., 2021). Briefly, a LAMP DNA amplification reaction [WarmStart^®^ LAMP Kit (DNA and RNA), NEB, Ipswich, MA, USA] was performed for cyclase gene amplification, containing 5 μL of extraction, 12.5 μL of WarmStart LAMP 2x Master Mix, and 2.5 μL of Primer Mix 10x (FIP/BIP 16 μM, F3/B3 2 μM, LOOPF/LOOPB 4 μM, stock 10X) adjusted to a final volume of 25 μL with RNase-free water and incubated at 65°C for 1 h. Following, a Leptotrichia wadei, LwaCas13a, endonuclease [GenCRISPR™ Cas13a (C2c2) Nuclease, GenScript] detection was assessed, using 2 μL of cleavage buffer 10X [GenCRISPR™ Cas13a (C2c2) Nuclease, GenScript], 0.5 μL of dNTPs (HiScribe™ T7 Quick High Yield RNA Synthesis Kit, NEB), 0.5 μL of T7 polymerase (HiScribe™ T7 Quick High Yield RNA Synthesis Kit, NEB), 20 U of RNase murine inhibitor (NEB), 25 nM of LwaCas13a endonuclease, 50 nM of crRNA (IDT), 1,000 nM of reporter (GenScript), and 5 μL of LAMP amplicon, adjusted to a final volume of 20 μL with RNase-free water and incubated at 37°C for 30 min (Table 4). Negative controls for both amplification and detection reactions were carried out using RNase-free water instead of sample. Finally, the test results were revealed using HybriDetect lateral flow test strips (Milenia Biotec, Giessen, Germany), mixing 20 μL of collateral cleavage activity-based detection product with 80 μL of assay buffer supplemented with 5% polyethylene glycol in a 96-well plate where strip tests were placed. The test results are visible to the naked eye after 2–3 min.

## Results

### PAIs and search analysis

**Figure 1:**
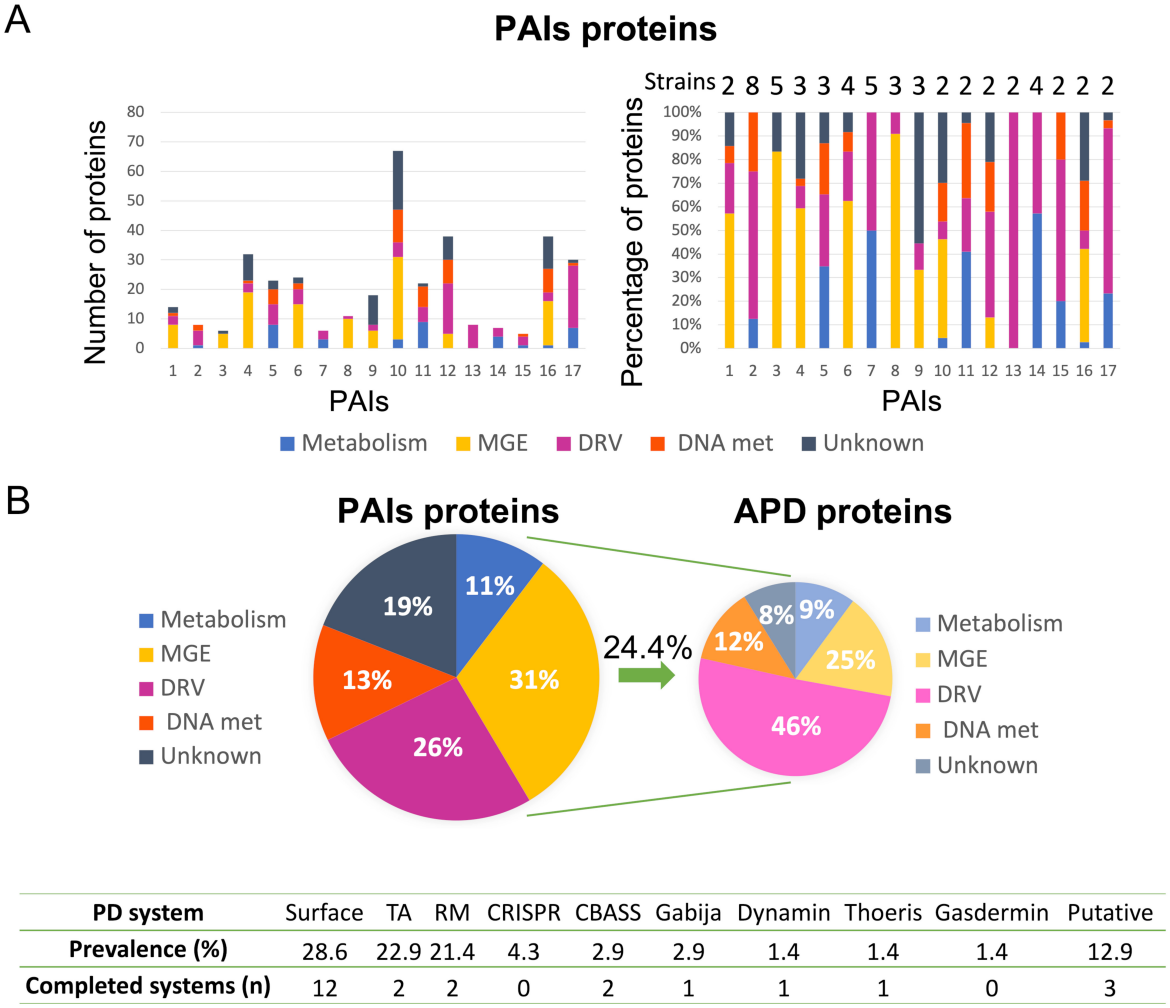
Results of the bioinformatic analysis of proteins contained in 17 *K. pneumoniae* PAIs. **(A)** Classification of proteins in functional groups, showing a number of proteins (left) and percentages of proteins (right) in each functional group of PAIs; the number of *K. pneumoniae* strains containing each PAI is also shown (right). **(B)** Global percentages of functional groups of proteins in PAIs (left); ADP proteins classified in the different functional groups (right); and a table containing the APD systems identified in these PAIs, their prevalence, and the number of the completed systems found (below).

Analysis of the genomes of 48 clinical isolates of *K. pneumoniae* led to the identification of a total of 309 regions with a high probability of being PAIs. Among these, 17 PAIs were detected in more than one *K. pneumoniae* strain, and the sequences were analyzed bioinformatically to determine the function of the proteins harbored in them, as greater spread could be related to important roles in bacterial fitness and adaptation (Figure 1A). The results showed that 31% of the proteins were related to MGE, 26% to DRV, 13% to DNA metabolism, 11% to metabolism, and 19% to unknown function. Furthermore, 24.4% of these proteins were involved in APD, and 46% of these corresponded to the DRV functional group, but also 25% to MGE, 12% to DNA metabolism, 9% to metabolism, and 8% were of unknown function. In particular, 90.5% of the APD proteins in MGE were proteins of phage origin. Finally, the organization of the APD proteins in the different APD systems revealed that most of these proteins are involved in surface modification (28.6%), TA (22.9%), and RM (21.4%) systems. We also identified several complete defense systems: 12 surface modifications, 2 TA, 2 RM, 2 CBASS, 1 Gabija, 1 dynamin, 1 Thoeris, and 3 putative systems (Figure 1B).

The complete genome of the *K. pneumoniae* strains with CBASS systems harbored in PAIs, 10ST340-VIM1 and K3325S were exhaustively analyzed by PADLOC, along with the genomes from the other 46 *K. pneumoniae* strains, to identify CBASS systems not contained in PAIs. The genomic structure of the CBASS systems in both 10ST340-VIM1 and K3325S strains was similar (Table 5), and only these two CBASS systems (11%) were located in PAIs (Table 6). Regarding the classification of the CBASS systems detected, 63% belonged to type I and 37% to type II. None of the type I CBASS systems were contained in PAIs, and two of the three type II CBASS systems were located in PAIs of the 10ST340-VIM1 and K3325S strains. Analysis of the components of these CBASS systems revealed that the cyclase APECO1 gene was present in 13 of the 15 (87%) type I CBASS systems detected but not in any of the type II CBASS systems. Regarding the effector protein, the pore-forming function effector appeared in all type I and in one of three type II (33%) CBASS systems; however, phospholipase and nuclease effectors were only located in type II CBASS systems (Table 6). Finally, the cyclic-nucleotide signal was GMPAMPc in all of the CBASS systems analyzed (Table 6).

**Table 5:** APD systems harbored in 10ST340-VIM1 and K3325S *K. pneumoniae* strains.

**System**	**Component**	**Positions**
**ST340-VIM1**		
PD-T4-3	PD-T4-3	1098241-1099020
cbass_other	Cyclase	1115885-1117165
cbass_other	E2	1117172-1117657
cbass_other	Effector	1117745-1118848
cbass_other	Effector	1119012-1119551
VSPR	Vsr	2787512-2788009
VSPR	MTase_II	2787987-2789387
PD-T7-1or5	PD-T7-1	3826360-3827727
SoFic	SoFic	4068190-4069191
AbiE	AbiEi	5058670-5059428
AbiE	AbiEii	5059434-5060351
DMS_other	DrmC	5422375-5422935
Shango	SngC	5426125-5428320
Shango	SngB	5428317-5429633
Shango	SngA	5429637-5431946
DMS_other	DrmD	5494251-5495036
DMS_other	DrmC	5497671-5498204
RM_type_II	MTase_II	5511251-5512684
RM_type_II	REase_II	5512718-5513926
**K3325S**		
cbass_other	Cyclase	1095759-1097039
cbass_other	E2	1097046-1097531
cbass_other	Effector	1097619-1098722
cbass_other	Effector	1098886-1099425
VSPR	Vsr	2944287-2944784
VSPR	MTase_II	2944762-2946162
retron_I-A	msr-msd	3542585-3542735
retron_I-A	RT_I-A	3542804-3543769
retron_I-A	ATPase_I-A	3543747-3545339
retron_I-A	HNH_I-A	3545336-3545965
DMS_other	DrmC	3547054-3547320
hachiman_type_I	HamB1	3547928-3550489
hachiman_type_I	HamA1	3550476-3552062
CRISPR_array	CRISPR_array	3703557-3706511
cas_type_I-E	Cas2e	3706608-3706901
cas_type_I-E	Cas1e	3706901-3707821
cas_type_I-E	Cas6e	3707818-3708468
cas_type_I-E	Cas5e	3708450-3709196
cas_type_I-E	Cas7e	3709207-3710262
cas_type_I-E	Cas11e	3710281-3710823
cas_type_I-E	Cas8e	3710820-3712376
cas_type_I-E	Cas3e	3712388-3715051
PD-T7-4	PD-T7-4	3920726-3921364
retron_II-A	NDT_II-A	4669058-4670008
retron_II-A	RT_II-A	4669995-4670945
RM_type_II	MTase_II	5477325-5478533
RM_type_II	REase_II	5478530-5479180

**Table 6:** Classification of CBASS systems contained in the 48 *K. pneumoniae* strains.

**Number**	**Name**	**CBASS system**	**APECO1 cyclase**
1	K3416	CBASS system I-B^GA^	Yes
2	K3325	CBASS system II-B^GA^	No
3	K3320	CBASS system I-B^GA^	No
4	K2791	CBASS system I-B^GA^	Yes
5	K2691	CBASS system I-B^GA^	Yes
6	K2597	CBASS system I-B^GA^	Yes
7	K2551	CBASS system I-B^GA^	Yes
8	K2535	CBASS system I-B^GA^	Yes
9	SCISP4C	No	
10	SCISP2C	No	
11	K3579	No	
12	K3575	No	
13	K3574	No	
14	K3573	No	
15	K3571	No	
16	K3509	No	
17	K3324	No	
18	K3323	No	
19	K3322	No	
20	K3321	No	
21	K2990	No	
22	K2989	No	
23	K2986	No	
24	K2984	No	
25	K2982	No	
26	K2707	No	
27	K3667	CBASS system I-B^GA^	Yes
28	K2715	No	
29	K2783	No	
30	K2983	No	
31	ST974-OXA48	No	
32	ST899-OXA48	No	
33	ST258-KPC3	No	
34	ST512-KPC3	No	
35	ST846-OXA48	No	
36	ST147-VIM1	No	
37	ST16-OXA48	No	
38	ST437-OXA245	No	
39	ST15-OXA48	CBASS system I-B^GA^	Yes
40	ST13-OXA48	CBASS system I-B^GA^	No
41	ST340-VIM1	CBASS system II-C^GA^	No
42	ST101-KPC2	CBASS system II-A^GA^	No
43	ST15-VIM1	CBASS system I-B^GA^	Yes
44	ST11-OXA245	CBASS system I	Yes
45	ST11-VIM1	CBASS system I-B^GA^	Yes
46	ST405-OXA48	No	
47	ST11-OXA48	CBASS system I-B^GA^	Yes
48	K3318	CBASS system I-B^GA^	Yes

A: Type I CBASS systems with a phospholipase as effector; B: type I CBASS systems with a pore-forming effector activity; C: type I CBASS systems with an endonuclease effector.

^GA^Cyclic GMP-AMP second messenger signal.

### Regulatory role of quorum sensing in CBASS system expression

**Figure 2:**
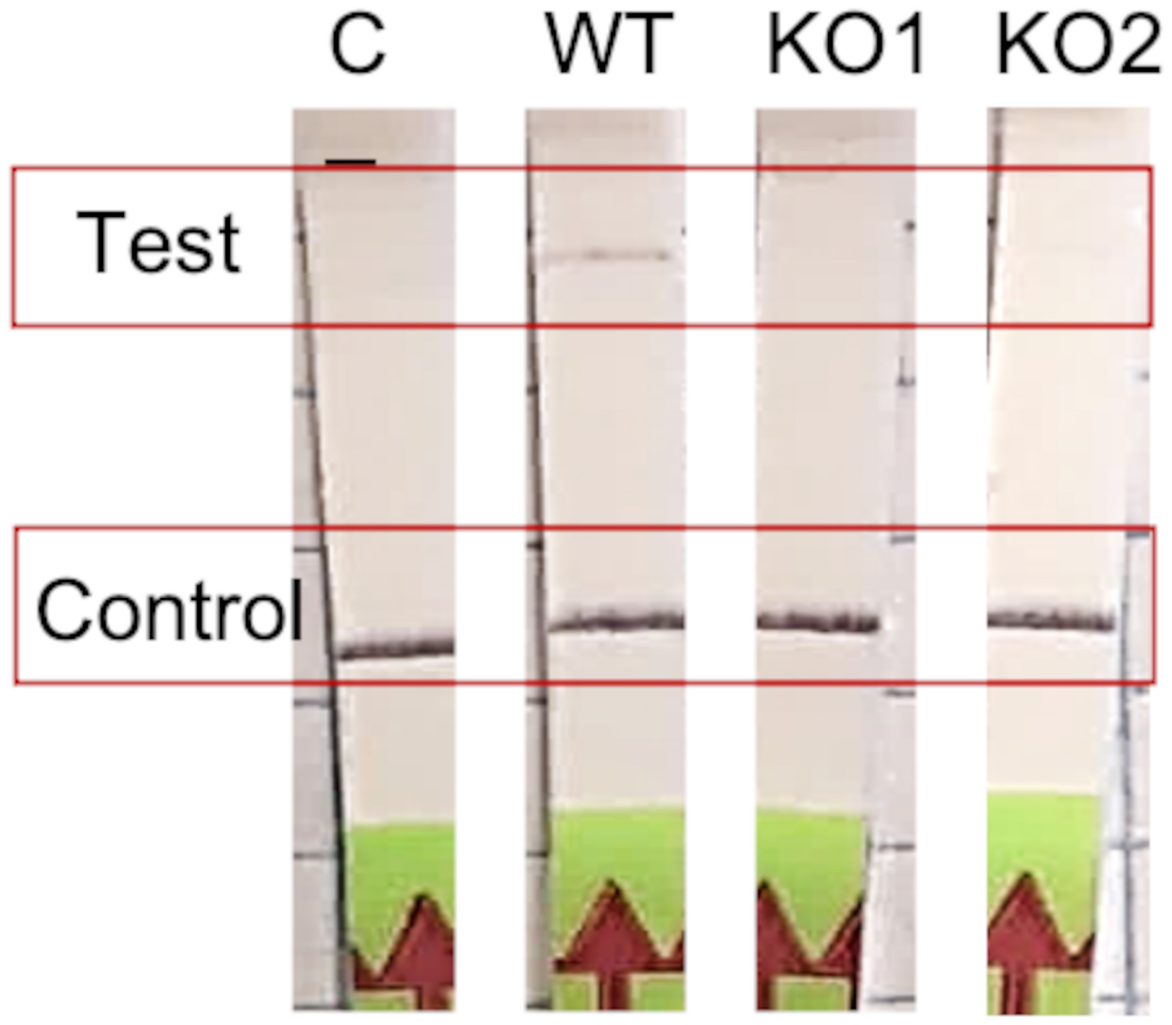
Results of the cyclase gene knockout construction by λ-Red protocol. Confirmation of the deletion of the cyclase gene in two mutants by LAMP-CRISPR-Cas13a protocol.

In order to study the regulatory role of QS in the expression of the type II CBASS system, we constructed a 10ST340-VIM1Δcyclase *K. pneumoniae* strain, and deletion of the gene was consecutively confirmed by the LAMP-CRISPR-Cas13a technology (Figure 2). A quantitative study of the type II CBASS system expression under QS activation conditions was conducted by measuring *effector I* gene expression levels in the presence of the AHL C6-HSL, in both 10ST340-VIM1 and 10ST340-VIM1Δcyclase *K. pneumoniae* strains. The results revealed overexpression of the APD system in the 10ST340-VIM1 *K. pneumoniae* strain (1.23) (Table 7). By contrast, the level of expression of the type II CBASS system in the 10ST340-VIM1Δcyclase *K. pneumoniae* strain (0.92) was similar to that of the *recA* housekeeping gene (Table 7).

**Table 7:** Quantitative expression of *effector I* gene by qRT-PCR.

	**Condition**	**Cp CBASS**
WT	Hex 20	1.23
KO	Hex 20	0.92

### Involvement of the PAI CBASS defense system in bacterial viability levels

**Figure 3:**
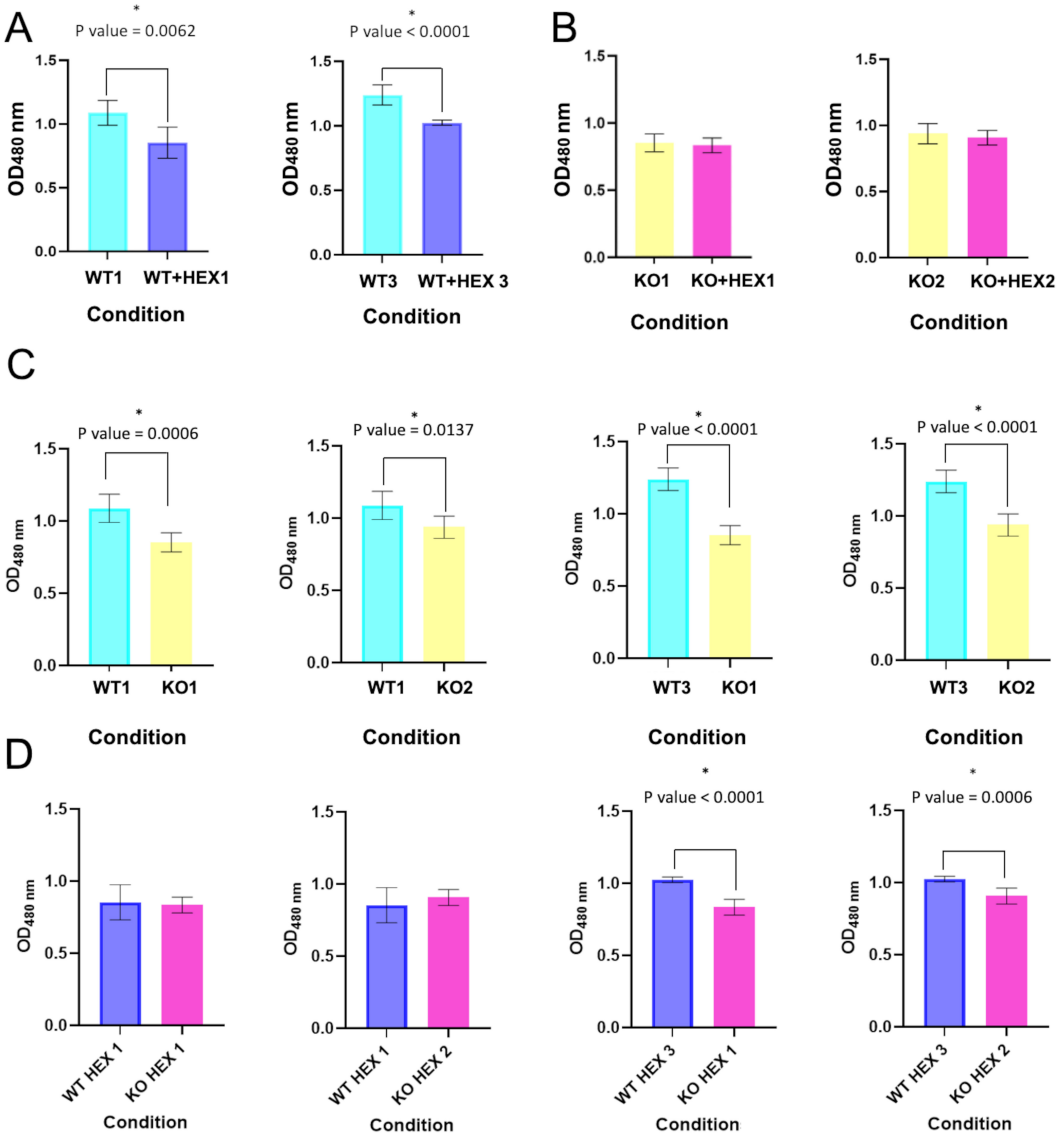
Cell viability in the presence and absence of AHL C6-HSL in both 10ST340-VIM1 and 10ST340-VIM1Δcyclase *K. pneumoniae* strains. **(A)** Comparison of cell viability in two 10ST340-VIM1 biological replicates. **(B)** Comparison of cell viability in two 10ST340-VIM1Δcyclase biological replicates. **(C)** Comparison of cell viability in the 10ST340-VIM1 and 10ST340-VIM1Δcyclase strains in the absence of AHL C6-HSL. **(D)** Comparison of cell viability in the 10ST340-VIM1 and 10ST340-VIM1Δcyclase strains in the presence of AHL C6-HSL. WT: 10ST340-VIM1 *K. pneumoniae* strain; KO: 10ST340-VIM1Δcyclase *K. pneumoniae* strain; HEX: AHL C6-HSL; Numbers on the condition axis refer to biological replicates. *Statistically significant difference.

The role of CBASS in bacterial viability was studied using an assay based on the WST-1 reagent. With this aim, cellular respiration levels in the 10ST340-VIM1 and 10ST340-VIM1Δcyclase *K. pneumoniae* strains were compared, using the AHL C6-HSL to induce the CBASS system. There was no significant difference in the viability of the 10ST340-VIM1Δcyclase *K. pneumoniae* strain with and without the autoinducer C6-HSL (Figure 3B), in contrast to the 10ST340-VIM1 *K. pneumoniae* strain, which showed a significant reduction in cell respiration when treated with AHL C6-HSL (Figure 3A). By contrast, the viability of the 10ST340-VIM1 *K. pneumoniae* strain was significantly higher than that of the 10ST340-VIM1Δcyclase *K. pneumoniae* strain (Figure 3C). Finally, the viability of both strains was very similar in the presence of AHL C6-HSL, except in one of the 10ST340-VIM1 *K. pneumoniae* strains that grew more than the other 10ST340-VIM1 and 10ST340-VIM1Δcyclase *K. pneumoniae* strains (Figure 3D).

### Detection by LAMP-CRISPR-Cas13a

**Figure 4:**
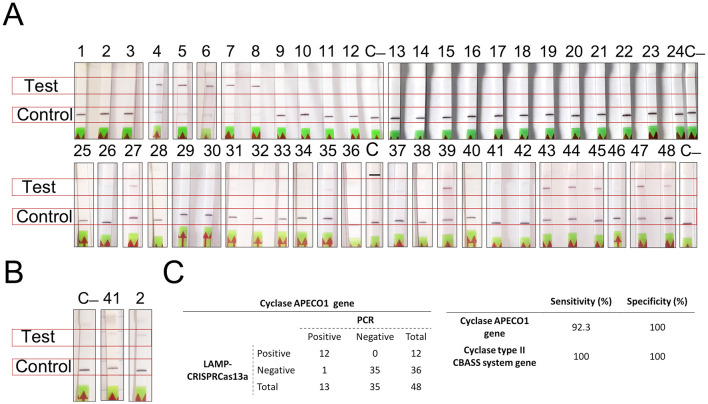
Results of the detection of type I and II CBASS systems using the LAMP-CRISPR-Cas13a technology. **(A)** Strips for detection of the *K. pneumoniae* strains harboring the APECO1 gene; **(B)** Strips for detection of the *K. pneumoniae* strains containing the cyclase gene in type II CBASS system; **(C)** APECO1 gene detection **(A)** and table showing the specificity and sensitivity of the LAMP-CRISPR-Cas13a technique, determined by processing data in **(A, B)**.

The LAMP-CRISPR-Cas13a technique produced very good results regarding the detection of the cyclase APECO1 present in several type I CBASS systems (*n* = 13) and the cyclase contained in the type II CBASS systems located in PAIs (*n* = 2). The technique was able to detect the cyclase APECO1 gene in all of the *K. pneumoniae* strains containing it, except strain number 1. It also produced negative results for the *K. pneumoniae* strains without CBASS systems and with type II CBASS systems and for the strains containing type I CBASS systems with a different cyclase (Figure 4A). Importantly, the application of a bioinformatic assay to the type I CBASS system in strain 1 indicated that the cyclase APECO1 located in this system had only seven nucleotides in common with the crRNA molecule, required for Cas13a detection. In addition, the technique successfully detected the presence of the cyclase in the type II CBASS systems contained in PAIs in the 10ST340-VIM1 and K3325S *K. pneumoniae* strains (Figure 4B). Based on the results obtained, we estimated that the technique exhibits 92.3% sensitivity and 100% specificity [99% confidence interval (CI)] for the detection of the cyclase APECO1 gene as well as 100% sensitivity and specificity [99% confidence interval (CI)] for the identification of the cyclase gene contained in type II CBASS systems located in PAIs (Figure 4C).

## Discussion

The results of the *in silico* analysis support the possibility that GIs are essential elements in the HGT for the optimization of bacterial fitness and adaptation, as more than 50% of the genes found in the 309 PAIs were MGE or were involved in DRV (Figure 1A). More than 20% of the proteins contained in the 309 PAIs were related to a single defense mechanism, that is, APD (Figure 1B; Fillol-Salom et al., 2022). This finding is consistent with those of Fillol-Salom et al. (2022), who demonstrated that PICIs are PAIs responsible for the transfer of defense genes.

A study of the complete APD systems harbored in the 17 most widespread PAIs revealed that these PAIs may be more important for bacterial fitness than previously considered, as the most prevalent anti-phage systems detected have a low cost to bacterial fitness (Figure 1B). Indeed, we did not detect any complete CRISPR-Cas system, in accordance with the increasingly prevalent idea that this APD system has a high cost for bacterial fitness, thus explaining its unexpectedly low prevalence across the bacterial domain (Zaayman and Wheatley, 2022). The cell surface modification and the defense systems harbored in prophages were notable findings, as preventing phage adsorption is the first line of defense in bacteria, and the main representative system. Prophages are widely spread and provide bacterial defense systems, which explains why almost all MGEs harboring APD proteins are prophages (Duan et al., 2024; Rousset et al., 2022). We also detected other common anti-phage systems: TA, RM, and CBASS APD systems. Abi systems have been detected in a wide variety of microorganisms, although their abundance is difficult to assess owing to the high level of diversity (Bernheim and Sorek, 2020).

Analysis of the CBASS systems contained in the 48 genomes of the *K. pneumoniae* strains identified almost twice as many type I CBASS systems as type II CBASS systems. Importantly, the fact that only type II CBASS systems were found in PAI sequences may indicate that this type of CBASS system is more important for bacterial fitness. This may also explain why the cyclase APECO1 is present in a high proportion of all type I CBASS systems (87%), while the presence of the cyclase involved in type II systems is more variable. According to the aforementioned explanation, the pore-forming effector function was homogeneously observed in the type I CBASS systems, but the effector harbored in the type II CBASS systems may be related to pore-forming or to phospholipase enzymatic function. Finally, the second signal GMPAMPc seems to be widespread as it is contained in all the CBASS systems detected.

Since being described in 2020 by Cohen et al., CBASS systems have been detected in the genomes of many prokaryotes, which demonstrates their widespread importance in enabling bacteria to resist phage replication (Cohen et al., 2019). The functional study of the type II CBASS system located in a PAI harbored in the 10ST340-VIM1 *K. pneumoniae* strain was assessed, due to the role of the PAIs in spreading resistance mechanisms and the fact that the 10ST340-VIM1 isolate contained fewer APD systems than the K3325S strain. The study focused on regulation by the QS network as well as on the role of the CBASS systems in the proliferation and cellular viability levels. The qRT-PCR assay showed that induction of the QS by AHL C6-HSL led to overexpression of the CBASS system in the 10ST340-VIM1 *K. pneumoniae* strain, indicating that detection of a threat to the bacterial community via the QS network provokes a signal that finally leads to activation of defense systems, some involved in anti-phage resistance, as previously described by Barrio-Pujante et al. (2024) (Figure 2). We also demonstrated that stimulation of the QS network causes a significant reduction in bacterial growth rates when the strain harbors a functional CBASS system, corroborating the aforementioned findings (Figure 3A), as CBASS is an Abi defense system. However, the lower growth rate of the 10ST340-VIM1Δcyclase *K. pneumoniae* strains suggests that the APD system may be involved in other mechanisms that improve bacterial fitness when growth conditions are suitable (Figure 3C).

Given the importance of novel therapeutic strategies like phage therapy, a reliable protocol for the rapid detection of bacterial resistance mechanisms, such as CBASS systems, is crucial for developing effective phage cocktails or combining these with other therapies to improve patient outcomes. We have demonstrated the value of the LAMP-CRISPR-Cas13a technique for detecting the most widespread CBASS types (I and II). This method has exhibited high sensitivity and specificity, except in detecting the cyclase APECO1 gene in type I CBASS systems. In this case, detection is impeded because one of 13 isolates harbors a modified version of the gene in the crRNA hybridization region, further highlighting the specificity of LAMP-CRISPR-Cas13a.

Building on our previous study, LAMP-CRISPR-Cas13a has proven highly effective for detecting bacterial resistance genes, including those conferring resistance to antibiotics and phages (Ortiz-Cartagena et al., 2023). The sensitivity and cost-effectiveness of the method are comparable to those of the molecular gold standard, RT-PCR (Ortiz-Cartagena et al., 2022, 2023). Furthermore, the high cost of RT-PCR, as well as the time requirements, and the need for specialized personnel and equipment make the method impractical for point-of-care (POC) testing, unlike CRISPR-Cas-based methods (Ortiz-Cartagena et al., 2022, 2023; Wu et al., 2021). Indeed, a CRISPR-Cas12a platform has been successfully used to detect β-lactamase-producing bacteria (KPC, NDM, and OXA), with a lower limit of detection (attomolar) than the CRISPR-Cas13a system (Curti et al., 2020; Wu et al., 2021). This may be because Cas12 targets double-stranded DNA (dsDNA), which is inherently more stable than RNA. However, the CRISPR-Cas12a protocol relies on RPA for isothermal amplification, which is less specific than LAMP, and uses a fluorescent readout, making it more costly than the LAMP-CRISPR-Cas13a method (Ortiz-Cartagena et al., 2022, 2023).

In summary, the study findings demonstrate the regulatory role of the QS network in the Abi system CBASS. Moreover, we propose an innovative biotechnological application for the LAMP-CRISPR-Cas13a technique as an ideal tool for optimizing phage therapy, as it could enable the detection of a particular APD mechanism, thus predicting the potential inefficacy of a therapeutic phage. The use of the LAMP-CRISPR-Cas13a technique is therefore proposed as a promising means of improving treatment selection and patient prognosis. Moreover, the study findings demonstrate the regulatory role of the QS network in the CBASS family of Abi systems.

## Data Availability

The raw data supporting the conclusions of this article will be made available by the authors, without undue reservation.

## References

[B1] Ajulo S. Awosile B. (2024). Global antimicrobial resistance and use surveillance system (GLASS 2022): investigating the relationship between antimicrobial resistance and antimicrobial consumption data across the participating countries. PLoS ONE 19:e0297921. 10.1371/journal.pone.0297921 38315668 PMC10843100

[B2] Audrey B. Cellier N. White F. Jacques P.-É. Burrus V. (2023). A systematic approach to classify and characterize genomic islands driven by conjugative mobility using protein signatures. Nucl. Acids Res. 51, 8402–8412. 10.1093/nar/gkad644 37526274 PMC10484663

[B3] Barrio-Pujante A. Bleriot I. Blasco L. Fernández-Garcia L. Pacios O. Ortiz-Cartagena C.. (2024). Regulation of anti-phage defense mechanisms by using cinnamaldehyde as a quorum sensing inhibitor. Front. Microbiol. 15:1416628. 10.3389/fmicb.2024.1416628 38989015 PMC11233531

[B4] Bernheim A. Sorek R. (2020). The pan-immune system of bacteria: antiviral defence as a community resource. Nat. Rev. Microbiol. 18, 113–119. 10.1038/s41579-019-0278-2 31695182

[B5] Bleriot I. Blasco L. Pacios O. Fernández-García L. López M. Ortiz-Cartagena C.. (2023b). Proteomic study of the interactions between phages and the bacterial host *Klebsiella pneumoniae*. Microbiol. Spectr. 11:e03974–e03922. 10.1128/spectrum.03974-22 36877024 PMC10100988

[B6] Bleriot I. Pacios O. Blasco L. Fernández-García L. López M. Ortiz-Cartagena C.. (2023a). Improving phage therapy by evasion of phage resistance mechanisms. JAC-Antimicrob. Resist. 6:dlae017. 10.1093/jacamr/dlae017 38343627 PMC10854218

[B7] Chen J. S. Ma E. Harrington L. B. Da Costa M. Tian X. Palefsky J. M.. (2018). CRISPR-Cas12a target binding unleashes indiscriminate single-stranded DNase activity. Science 360, 436–439. 10.1126/science.aar6245 29449511 PMC6628903

[B8] Cohen D. Melamed S. Millman A. Shulman G. Oppenheimer-Shaanan Y. Kacen A.. (2019). Cyclic GMP–AMP signalling protects bacteria against viral infection. Nature 574, 691–695. 10.1038/s41586-019-1605-5 31533127

[B9] Curti L. A. Pereyra-Bonnet F. Repizo G. D. Fay J. V. Salvatierra K. Blariza M. J.. (2020). CRISPR-based platform for carbapenemases and emerging viruses detection using Cas12a (Cpf1) effector nuclease. Emerg. Microb. Infect. 9, 1140–1148. 10.1080/22221751.2020.1763857 32486913 PMC7448918

[B10] Díaz M. L. (2017). Estudio del “quorum sensing” en “Acinetobacter baumannii”: *Implicaciones Cl*í*nicas* (eds. M. M. Tomás Carmona and G. Bou Arévalo). Programa de Doctorado en Ciencias de la Salud (RD99/2011). Instituto de Investigación Biomédica (INIBIC). Coruña: Complejo Hospitalario Universitario A Coruña- Universidad de A Coruñaeds.

[B11] Dobrindt U. Hochhut B. Hentschel U. Hacker J. (2004). Genomic islands in pathogenic and environmental microorganisms. Nat. Rev. Microbiol. 2, 414–424. 10.1038/nrmicro884 15100694

[B12] Doron S. Melamed S. Ofir G. Leavitt A. Lopatina A. Keren M.. (2018). Systematic discovery of antiphage defense systems in the microbial pangenome. Science 359:eaar4120. 10.1126/science.aar4120 29371424 PMC6387622

[B13] Duan N. Hand E. Pheko M. Sharma S. Emiola A. (2024). Structure-guided discovery of anti-CRISPR and anti-phage defense proteins. Nat. Commun. 15:649. 10.1038/s41467-024-45068-7 38245560 PMC10799925

[B14] Fillol-Salom A. Rostøl J. T. Ojiogu A. D. Chen J. Douce G. Humphrey S.. (2022). Bacteriophages benefit from mobilizing pathogenicity islands encoding immune systems against competitors. Cell 185, 3248–3262.e20. 10.1016/j.cell.2022.07.014 35985290

[B15] Finn R. D. Clements J. Eddy S. R. (2011). HMMER web server: interactive sequence similarity searching. Nucleic Acids Res. 39, W29–37. 10.1093/nar/gkr367 21593126 PMC3125773

[B16] Genoud V. Stortz M. Waisman A. Berardino B. G. Verneri P. Dansey V.. (2021). Extraction-free protocol combining proteinase K and heat inactivation for detection of SARS-CoV-2 by RT-qPCR. PLoS ONE 16:e0247792. 10.1371/journal.pone.0247792 33635936 PMC7909620

[B17] Georjon H. Bernheim A. (2023). The highly diverse antiphage defence systems of bacteria. Nat. Rev. Microbiol. 21, 686–700. 10.1038/s41579-023-00934-x 37460672

[B18] Gomes A. É. I. Stuchi L. P. Siqueira N. M. G. Henrique J. B. Vicentini R. Ribeiro M. L.. (2018). Selection and validation of reference genes for gene expression studies in Klebsiella pneumoniae using Reverse Transcription Quantitative real-time PCR. Sci. Rep. 8:9001. 10.1038/s41598-018-27420-2 29899556 PMC5998039

[B19] Grundmann H. Glasner C. Albiger B. Aanensen D. M. Tomlinson C. T. Andrasević A. T.. (2017). Occurrence of carbapenemase-producing *Klebsiella pneumoniae* and *Escherichia coli* in the European survey of carbapenemase-producing Enterobacteriaceae (EuSCAPE): a prospective, multinational study. Lancet Infect. Dis. 17, 153–163. 10.1016/S1473-3099(16)30257-2 27866944

[B20] Huang T.-W. Lam I. Chang H.-Y. Tsai S.-F. Palsson B. O. Charusan P. (2014). Capsule deletion via a λ-Red knockout system perturbs biofilm formation and fimbriae expression in *Klebsiella pneumoniae* MGH 78578. BMC Res. Notes 7:13. 10.1186/1756-0500-7-13 24398052 PMC3892127

[B21] Humphrey S. San Millán Á. Toll-Riera M. Connolly J. Flor-Duro A. Chen J.. (2021). Staphylococcal phages and pathogenicity islands drive plasmid evolution. Nat. Commun. 12:5845. 10.1038/s41467-021-26101-5 34615859 PMC8494744

[B22] Jonas O. B. Irwin A. Berthe F. C. J. Le Gall F. G. Marquez P. V. (2017). Drug-resistant infections : a threat to our economic future (Vol. 2 of 2): final report (English). HNP/Agriculture Global Antimicrobial Resistance Initiative. Washington, DC: World Bank Group. Available online at: http://documents.worldbank.org/curated/en/323311493396993758

[B23] Kellner M. J. Koob J. G. Gootenberg J. S. Abudayyeh O. O. Zhang F. (2019). SHERLOCK: nucleic acid detection with CRISPR nucleases. Nat. Protoc. 14, 2986–3012. 10.1038/s41596-019-0210-2 31548639 PMC6956564

[B24] Leamon J. H. Rothberg J. M. (2009). “DNA sequencing and genomics,” in Encyclopedia of Microbiology, 3rd Edn, ed. M. Schaechter (Academic Press), 148–161. 10.1016/B978-012373944-5.00024-9

[B25] Lin D. M. Koskella B. Lin H. C. (2017). Phage therapy: an alternative to antibiotics in the age of multi-drug resistance. World J. Gastrointest. Pharmacol. Ther. 8, 162–173. 10.4292/wjgpt.v8.i3.162 28828194 PMC5547374

[B26] Millman A. Melamed S. Amitai G. Sorek R. (2020). Diversity and classification of cyclic-oligonucleotide-based anti-phage signalling systems. Nat. Microbiol. 5, 1608–1615. 10.1038/s41564-020-0777-y 32839535 PMC7610970

[B27] Murray C. J. L. Ikuta K. S. Sharara F. Swetschinski L. Robles Aguilar G. Gray A.. (2022). Global burden of bacterial antimicrobial resistance in 2019: a systematic analysis. Lancet 399, 629–655. 10.1016/S0140-6736(21)02724-0 35065702 PMC8841637

[B28] Oliveira P. H. Touchon M. Rocha E. P. C. (2016). Regulation of genetic flux between bacteria by restriction-modification systems. Proc. Natl. Acad. Sci. U. S. A. 113, 5658–5663. 10.1073/pnas.1603257113 27140615 PMC4878467

[B29] Ortiz-Cartagena C. Fernández-García L. Blasco L. Pacios O. Bleriot I. López M.. (2022). Reverse transcription-loop-mediated isothermal amplification-CRISPR-Cas13a technology as a promising diagnostic tool for SARS-CoV-2. Microbiol. Spectr. 10:e0239822. 10.1128/spectrum.02398-22 36169448 PMC9604158

[B30] Ortiz-Cartagena C. Pablo-Marcos D. Fernández-García L. Blasco L. Pacios O. Bleriot I.. (2023). CRISPR-Cas13a-based assay for accurate detection of OXA-48 and GES carbapenemases. Microbiol. Spectr. 11:e0132923. 10.1128/spectrum.01329-23 37466441 PMC10434040

[B31] Pacheco T. Gomes A. É. I. Siqueira N. M. G. Assoni L. Darrieux M. Venter H.. (2021). SdiA, a quorum-sensing regulator, suppresses fimbriae expression, biofilm formation, and quorum-sensing signaling molecules production in *Klebsiella pneumoniae*. Front. Microbiol. 12:597735. 10.3389/fmicb.2021.597735 34234747 PMC8255378

[B32] Pacios O. Blasco L. Bleriot I. Fernandez-Garcia L. González Bardanca M. Ambroa A.. (2020). Strategies to combat multidrug-resistant and persistent infectious diseases. Antibiotics 9:65. 10.3390/antibiotics9020065 32041137 PMC7168131

[B33] Payne L. J. Meaden S. Mestre M. R. Palmer C. Toro N. Fineran P. C.. (2022). PADLOC: a web server for the identification of antiviral defence systems in microbial genomes. Nucleic Acids Res. 50, W541–W550. 10.1093/nar/gkac400 35639517 PMC9252829

[B34] Ramsay J. Colombi E. Terpolilli J. Ronson C. (2023). “Symbiosis islands,” in Reference Module in Life Sciences (Amsterdam: Elsevier). 10.1016/B978-0-12-822563-9.00071-8

[B35] Reyes J. Aguilar A. C. Caicedo A. (2019). Carbapenem-resistant *Klebsiella pneumoniae*: microbiology key points for clinical practice. IJGM Volume 12, 437–446. 10.2147/IJGM.S214305 31819594 PMC6886555

[B36] Rousset F. Depardieu F. Miele S. Dowding J. Laval A.-L. Lieberman E.. (2022). Phages and their satellites encode hotspots of antiviral systems. Cell Host Microbe. 30, 740–753.e5. 10.1016/j.chom.2022.02.018 35316646 PMC9122126

[B37] Seemann T. (2014). Prokka: rapid prokaryotic genome annotation. Bioinformatics 30, 2068–2069. 10.1093/bioinformatics/btu153 24642063

[B38] Singh K. Biswas A. Chakrabarti A. K. Dutta S. (2023). Phage therapy as a protective tool against pathogenic bacteria: how far we are? Curr. Pharm. Biotechnol. 24, 1277–1290. 10.2174/1389201024666221207114047 36503459

[B39] Soares S. C. Geyik H. Ramos R. T. J. De Sá P. H. C. G. Barbosa E. G. V. Baumbach J.. (2016). GIPSy: genomic island prediction software. J. Biotechnol. 232, 2–11. 10.1016/j.jbiotec.2015.09.008 26376473

[B40] Söding J. Biegert A. Lupas A. N. (2005). The HHpred interactive server for protein homology detection and structure prediction. Nucleic Acids Res. 33, W244–248. 10.1093/nar/gki408 15980461 PMC1160169

[B41] Tamura K. Stecher G. Kumar S. (2021). MEGA11: molecular evolutionary genetics analysis version 11. Mol. Biol. Evol. 38, 3022–3027. 10.1093/molbev/msab120 33892491 PMC8233496

[B42] Tang K. W. K. Millar B. C. Moore J. E. (2023). Antimicrobial resistance (AMR). Br. J. Biomed. Sci. 80:11387. 10.3389/bjbs.2023.11387 37448857 PMC10336207

[B43] Wang J. Chitsaz F. Derbyshire M. K. Gonzales N. R. Gwadz M. Lu S.. (2023). The conserved domain database in 2023. Nucleic Acids Res. 51, D384–D388. 10.1093/nar/gkac1096 36477806 PMC9825596

[B44] Wang L. Zhang L. (2023). The arms race between bacteria CBASS and bacteriophages. Front. Immunol. 14:1224341. 10.3389/fimmu.2023.1224341 37575224 PMC10419184

[B45] WHO (2024). WHO Bacterial Priority Pathogens List Bacterial Pathogens of Public Health Importance, to Guide Research, Development, and Strategies to Prevent and Control Antimicrobial Resistance. Geneva: World Health Organization.

[B46] Wu Y. Battalapalli D. Hakeem M. J. Selamneni V. Zhang P. Draz M. S.. (2021). Engineered CRISPR-Cas systems for the detection and control of antibiotic-resistant infections. J. Nanobiotechnol. 19:401. 10.1186/s12951-021-01132-8 34863214 PMC8642896

[B47] Zaayman M. Wheatley R. M. (2022). Fitness costs of CRISPR-Cas systems in bacteria. Microbiology 168:1209. 10.1099/mic.0.001209 35849532

[B48] Zahra A. Shahid A. Shamim A. Khan S. H. Arshad M. I. (2023). The SHERLOCK platform: an insight into advances in viral disease diagnosis. Mol. Biotechnol. 65, 699–714. 10.1007/s12033-022-00625-7 36494593 PMC9735230

